# Hospital Real-Time Location System (A Practical Approach in Healthcare): A Narrative Review Article

**Published:** 2019-04

**Authors:** Leila GHOLAMHOSSEINI, Farahnaz SADOUGHI, Aliasghar SAFAEI

**Affiliations:** 1. Department of Health Information Management, School of Health Management and Information Sciences, Iran University of Medical Sciences, Tehran, Iran; 2. Department of Health Information Technology, School of Paramedical Sciences, AJA University of Medical Sciences, Tehran, Iran; 3. Health Management and Economics Research Center, School of Health Management and Information Sciences, Iran University of Medical Sciences, Tehran, Iran; 4. Department of Biomedical Informatics, School of Medical Sciences, Tarbiat Modarres University, Tehran, Iran

**Keywords:** Hospital real-time location system (HRTLS), Radio-frequency identification (RFID), Global positioning system (GPS), Wi-Fi, Iran

## Abstract

**Background:**

The Hospital Real-time Location Systems (HRTLS), deal with monitoring the patients, medical staff and valuable medical equipment in emergency situations. Therefore, the study aimed to propose Hospital Real-Time Location Systems based on the novel technologies in Iran.

**Methods:**

In this narrative-review, the articles and official reports on HRTLS, were gathered and analyzed from related textbooks and indexing sites with the defined keywords in English or Persian. The search of databases such as IDTechEx, IEEE, PubMed Central, Science Direct, EMBASE/Excerpta Medica, Scopus, Web of Science, Elsevier journals, WHO publications and Google Scholar was performed to reconfirm the efficiency of HRTLS from 2006 to 2017.

**Results:**

Various technologies have been used in the current systems, which have led to the reduced error rate, costs and increased speed of providing the healthcare services. Applications of these systems include tracking of patient’s, medical staff and valuable medical assets. Besides, achieving the patient & staff satisfaction is among other basic applications of these Systems. The accurate data exchange and processes control are considered as positive aspects of this technology.

**Conclusion:**

HRTLS has great importance in healthcare systems and its efficiency in medical centers is reliable; hence, it seems necessary to determine the organization’s requirements, apply novel technologies such as cloud computing and Internet of things, and integrate them to get access to maximum advantages in Iranian healthcare centers.

## Introduction

The importance of people and objects' location in the healthcare centers is expanding quickly in recent years (1). On the other hand, one of the main problems at the time of crisis is an increase in the number of injured patients requiring emergency care. In such conditions, the hospitals might face various constraints such as shortage of resources, hospital beds, and health care personnel (2). Moreover, the lack of sufficient supervisory tools, which can instantly monitor the location of patients, physicians, medical staff, and medical assets and provide them for the hospital managers, has resulted in the loss of numerous opportunities (3). Today, development of the newly emerging intelligence systems considerably influences different aspects of life, one of the fields of health. The position of health in future perspective of the countries necessitates the hospitals to exploit advanced information technologies to automate the process (4). The most important issue in a hospital is the real-time tracking of physical position of the entities; therefore, it is essential to design and implement an appropriate Hospital Real-time Location Systems (HRTLS) for healthcare centers (5).

“HRTLS, an application based on ubiquitous computing, is recognized that will increase the visibility and operational efficiency of clinical and administrative workflows in the healthcare setting. Implementing an RTLS-based system in a hospital is an outstanding technology which can enhance healthcare system efficiency and automation” (6).

The application of RTLS in the field of healthcare is that, in case of occurrence of medical emergency for patients, the health care staff can be located quickly. This system can be also used to track the patients’ movement and ensure their safety, especially patients with Alzheimer and dementia (1). Nowadays, hospital real-time location systems propose many opportunities in healthcare systems for managing hospital services. Some of the most important applicable objectives the HRTLS model include real-time location of patient & physician workflow, increased patient/provider safety, monitoring the valuable hospital equipment such as medical asset temperature monitoring, bed capacity management, equipment maintenance, patient elopement and other beneficial applications (7). In general, these systems are divided into two intra-organizational and extra-organizational groups. The extra-organizational systems require broader coverage and are realized via various technologies such as satellite location. Global Positioning System (GPS) in extra-organizational space was used as real-time patient tracking software, but it could not achieve any significant success in the intra-organizational space (8).

The present study aimed to recognize the current status and investigate performance of hospital real-time location systems in order to determine the functional and non-functional requirements and propose a novel technology to get access to maximum advantages.

## Methods

In this narrative review article, an initial screening of publications such as books, articles, guides and standards, theses or dissertations, and reports of the governmental organizations or IT-related companies based on titles, was performed by three researchers. In the second screening round of the remaining publications, titles and abstracts were evaluated by reviewers independently. All articles published in indexing sites with the defined keywords in English or Persian were gathered. The keywords were as follows: (“Hospital Real-Time Location System” or “HRTLS”), (“Radio-Frequency Identification” or “RFID”), (“Global Positioning System” or “GPS”), (“Wireless Local Area Network” or “WLAN”), (“Access Points” or “AP”), Indoors/Outdoors Positioning, Wi-Fi, (“Cloud computing” or “Internet of things” or “IOT” or “CloudIOT” or “Cloud of things”) and (“Iran” or “providing” or “establishing”). The indexing websites included IDTechEx, IEEE, PubMed Central, Science Direct, Elsevier journals, WHO publications and Google Scholar. The authors attempted to study some HRTLS samples using the RFID systems in countries such as Australia, Belgium, Canada, Germany, Iran, Italy, Japan, Luxembourg, Mexico, Netherlands, Russia, South Korea, Turkey, United Kingdom (UK) and The United States of America (USA) respectively.

The study population included the documents and resources related to HRTLS, which qualified the following criteria. The first criterion was the extraction and entry of the resources and their thematic relevance with the research objective; accordingly, the resources containing the HRTLS-related information were investigated. The second criterion was the access to abstract or full text of the retrieved resources; besides, the third criterion was language of the resource, considered as Persian or English. The resources lacking these criteria were excluded from the study. Papers were excluded based on the following criteria:

- Published before 2006 (if describing organization or financing of hospital real-time location system)
- Not relevant to the title of study
- Published in languages other than English or Persian
- Not relevant to specific countries
- Not in line with the working definition

After final selection of the papers, information was extracted from full texts. Other documents included related books and regulations. Among 148 retrieved sources (19 in Persian and 129 in English), 44 sources were chosen based on complete relevance, originality and expertness of the authors in providing hospital real-time location system. Sensitive search for controlling publication bias was done. Limitation of the study was that very few papers in the literature and documentations were Iranian, so Iranian literature were less.

## Results

HRTLS were investigated using various objectives in the 15 selected countries. The Radio Frequency Identification (RFID) Knowledgebase is an online searchable database, that all studies on hospital real-time location systems from all around the world have been recorded (9–12).

The results of this study can be explained in eight categories:

### a) Elderly care tracking systems

The population of elderly people is increasing rapidly in many developed nations. Providing safe and comfortable care for aging people is an important social goal. Moreover, obtaining correct activity and real-time location information for an elderly person is very important. In 2011, a novel was proposed intelligent RFID-based indoor tracking system for elderly persons. The proposed system used environment information for inhabitants and received signal strength of an RFID reader to estimate the probable location of an inhabitant. The accuracy of the proposed system is better than that of existing RFID-based systems (13).

On the other hand, an indoor U-Healthcare system used radio-frequency identification technology to accurately locate and track the elderly, and provide the real-time monitoring of elderly people’s whereabouts (14).

Before that in Australia, PresCare Company used a Wi-Fi-based RFID system. PresCare had service offices and communication centers, which used active tags, hardware, and Aero Scout software. In this system, about 100 tags sent their identification number to the closest AP, and then this information was sent to the display screens installed in the monitoring rooms (15).

### b) Medical asset tracking systems

A system for identification, tracking, and management of medical assets is a vital need for every hospital. A Belgium hospital implemented the RFID-based RTLS system. The purpose of this system was to locate the patients and hospital assets in emergency conditions. The hospital sent the signals to the hospital’s Wi-Fi network through active tags; then, a tag was allocated to each medical assets, which included the information that could be seen via text messages in the nurses’ cell phones (16). Furthermore, St. Louis Hospital in Luxemburg selected the Aero Scout solutions and used the active Wi-Fi-based RFID tags; therefore, using the RFID readers led to time-saving and reduced costs. This system can help improve patient care and reduce costs (17). Moreover, in general, Mexico hospital, the WLAN infrastructure was applied using Cisco products, and the information was sent from the tags to Aero Scout software (18). TerGooi hospital in Netherlands is another hospital that uses this system. Some of the advantages of this system include the optimal use of time for staff and provision of better care services for patients (19). Finally, an RFID network design methodology was performed for asset tracking in healthcare systems and proposed a new methodology to optimize the medical-asset tracking system by RFID readers (20).

### c) Children and elderly security systems

Continuous monitoring is necessary in healthcare systems and helps to caregivers in order to secure chronic patients, elderly people, and children (21). For this purpose, Canadian ProSolutions Company to implement an RFID-based HRTLS system in order to protect the infants and the patients with Alzheimer. In this system, the Blue Tag software embedded in a wristband uses active RFID tags. The alarm bell will ring up in case the person equipped with these tags passes through the exit doors, equipped with readers (18). Besides, X-tag Company experimentally created a new wristband for patient’s security systems. When patients leave the hospital, readers will automatically send an alert message to security staff and hospital nurses to determine exact position of the patient's (22).

### d) Blood transfusion monitoring systems

**Fig. 1:**
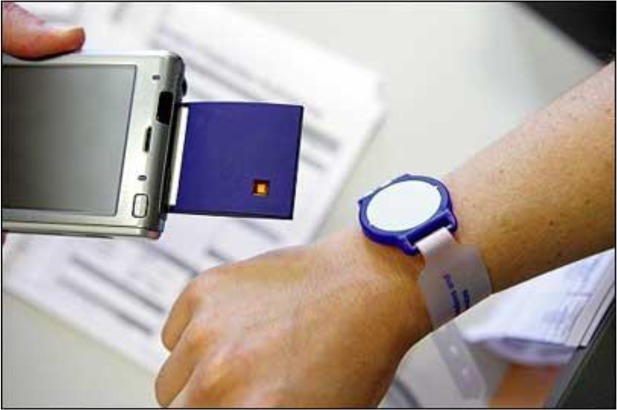
RFID-based wristbands

Blood transfusion is a necessity for trauma patients; therefore, automated prediction of early blood transfusion is very important for decrease mortality rate of patients (23). In this regard, a research project was presented to assess the availability of blood reserve, tracking and management system (BRAMS). The current implementation of the system is used by 130 users at different levels in the supply chain in Turkey (24). Winterberg hospital used RFID tags for injection of blood, which led to reduced errors in the blood injection process. In this hospital, one thousand blood packs were marked and then the injection stages were recorded. Inside the patient's wristband, there was a passive RFID electronic chip. The patient's and the blood bag data must be matched before the blood can be used, because of minimizing the risk of receiving the wrong type of blood (25). On the other hand, a pilot study in the Italian National Central Institution was performed to increase the management efficiency in transfer of the blood products. In this plan, the RFID tags on blood packs as well as the patient’s RFID-based wristbands (Fig. 1) were used (26).

### e) Medication tracking systems

“Medication tracking systems is one of the most important factors that directly affect the quality of health care. Minimizing the occurrence of adverse events is one of the main challenges for health professionals. The systems use RFID technology to perform various tasks: 1) locate patients in different areas; 2) measure patient care times and waiting times; 3) identify unitary doses of medication, and 4) ensure the correct matching between the patient and the medication prescribed by the physician” (27).

Furthermore, a new medication tracking system in Germany was tested using RFID. This plan was primarily launched to control the consumption of antibiotics. Before prescription, the pharmacist installed a tag on the medicine package, and then the readers read the tag information at the time of drug withdrawal from pharmacy and transferred the patient’s status to the server (28).

Then, in Novartis Pharmaceuticals Company, the active tags were used experimentally in drug packs to track the blood pressure drug Novartis. When the patient returned the empty medicine pack to the pharmacy, the information was sent to the central database. This method, which monitored consistent use of prescription drug, led to the proper use of the drug program by patients (29).

### f) Patient’s tracking system

According to an article published in IEEE Xplore digital library, “Health monitoring systems have rapidly evolved recently, and smart systems have been proposed to monitor patient current health conditions. The system will track, trace, monitor patients and facilitate taking care of their health; so efficient medical services could be provided at appropriate time” (30).

Hence, Trevigilo-Caravaggio Italian hospital implemented RFID system to track emergency patients. Once admitted, the new patients received an active RFID tag, and each tag had an RFID number connected to the hospital database, which included the patient’s name and health information. Meanwhile, the hospital portals were equipped with RFID readers for real-time location of the patients (31). Moreover, Michigan Oakwood Hospital used Aero Scout's Visibility Solution to track the patient’s location. In this system, Wi-Fi Based Active RFID tags were used, which sent the information to the Wi-Fi network wireless infrastructure (32).

RTLS “LLC” is a Russian company, which monitors the location of people and objects with high precision by providing hardware and software technologies. “LLC” designs these systems with higher reliability and stability (33). In the same field, RTLS “LLC” designed a high-performance and survivability system, which determines the location of people and objects in real-time and has been working even during power outage or failure of individual elements. This system manages all vital components, calculates the patient/provider coordinates, creates a graphical representation of the objects flow, and provides reports for the external users of the systems (34).

On the other hand, An Emergency Center in America used RTLS to improve its performance in patient care. Upon the admission, a wristband with RFID tag was given to the patient. The software visualizes patient flow and identifies the location of patients and medical equipment (35).

A combination of the RFID systems and WLAN communicational network in South Korea was used for patient tracking; accordingly, at the time of admission, an Aero Scout T2 tag was given to each patient. Using this software, the staff could observe the patients’ location on the map, and follow the pathway or traveled route (36). Moreover, inpatient tracking system at Birmingham hospital, RFID wristbands at HF and passive frequency band used on which the identification number, name, date of birth, and sex of the patient were encoded. Besides, the patient’s electronic image was sent to the hospital’s computer system. The wristbands were scanned, and when the wristband information was read once, the hospital system could prevent re-registration of the patient’s information (22). Another healthcare center, American St. Vincent’s, had an RFID-based system launched as a preliminary project for cardiovascular patients. The RFID readers were connected wirelessly to the hospital’s Ethernet network and sent the patient’s spatial information to a Structured Query Language (SQL) database (37). In 2012, 23 U.S. hospitals were investigated. Investigations on the HRTLS systems focused on the type of the applied RTLS, different software capabilities and technologies of this system (38).

In the other project, a Wireless Body Area Sensor Networks (WBASN) was used wireless sensors to implement real-time monitoring of patients. This system proposes a prototype of cloud mobile health monitoring system and uses WBASN and Smartphone applications such as cloud computing, location data, and a neural network to determine the state of patients (39).

### g) Real-time data collection systems

The Point of Act System (POAS) international drug center in Japan was formed operationally, which was a real-time data collection system. This system gathers the data; in fact, it deals with answering questions such as When, Where and Who. By real-time collection of data from Personal Digital Assistant (PDA), the terminals affiliated to the laboratory and POAS laboratory equipment can record the medical operation details anywhere and monitor the patient’s disease symptoms or recovery symptoms continuously (40).

### h) Monitoring healthcare environments

HamiTag Iranian Company provides real-time monitoring of the healthcare environments using HRTLS, and is one of the best systems that provide various tags and powerful Active RFID readers; therefore, the data exchange occurs in a completely secure environment without the slightest impact on wireless systems. Based on this strategy, a wireless communication platform based on an encrypted protocol gathers the data continuously and without any interference with other communication platforms (41).

## Discussion

In healthcare facilities, HRTLS can be used to locate portable assets, equipment management and reducing the need for inventory, monitoring the location of patients & providers in real or near real-time, enhancing patient safety, work-flow management during a medical emergency, improving staff utilization, and reducing error in information delivery. In the present study, the selected countries were investigated in terms of type of equipment, working frequency, type of use, and purpose of using HRTLS such as elderly care tracking system, medical asset tracking system, children and elderly security system, blood transfusion monitoring system, medication tracking system, patient’s tracking system, real-time data collection system, and monitoring healthcare environments in medical centers.

Due to the influential role of various factors such as accuracy, reliability, speed, security, and safety on providing care services for patients, as well as direct affectability of the patients from inevitable mistakes of the medical staff, it seems essential to develop hospital real-time location system in healthcare systems. Therefore, the use of applicable system would promote the quality of healthcare services and, as a result, lead to the increased patient satisfaction level (42).

According to healthcare provider predictions, “RTLS market worth would reach to 8.09 Billion USD by 2022”. Today, many hospitals around the world are paying a great deal of money to replace lost medical equipment and monitoring hospital resources, patients, and medical staff; Therefore, to solve problems in the field of patient monitoring, medical staff and valuable medical equipment, the RTLS solution offers a golden opportunity to hospitals to maximize benefits with minimal cost and improve hospital work-flow process (43). Most of the intelligence systems rely on radio-frequency communications for patient identification and tracking (11–16, 18–20, 25–29, 31); furthermore, in case of crisis or emergency situations, these traditional systems encounter considerable constraints, which result in low-quality services (44).

Serious barriers were revealed in technology, infrastructure, and organizational obstacles in the application of HRTLS; therefore, in order to maximize the potential benefits of the system, these barriers should be eliminated as soon as possible (39). Many of the currently existing RTLS systems in medical centers use technologies such as RFID, WLAN, Wi-Fi, and Bluetooth; Moreover, in some of medical centers, other technologies such as RFID tag wristbands, Sonitor’s IPS active tags, Aero Scout Software, Wi-Fi-based Active RFID tags and Passive RFID are used as well (15,18,35,50).

Today’s competitive era in healthcare market is increasing the pressure on enterprises that in turn driving the adoption of HRTLS technologies. The increased adoption of cost-effective technologies like ZigBee and UWB, ultrasound and infrared (IR) technologies, and growing smart hospital in the healthcare sector, are some of the key trends identified in the hospital systems. Some of the main constraints of this system are lack of interoperability between infrastructures, privacy concerns, higher cost of implementation and maintenance of the system (45).

According to a report in the global real-time location system market, “the RTLS market has been segmented into North America, Europe, Asia-Pacific, Latin America, and the Middle East & Africa. Asia-Pacific is projected to lead the RTLS market with the highest growth rate during the forecast period, due to the increasing awareness of advantages offered by RTLS and the escalating demand from the growing industrial sector” (45). The North American, a technology-based country, which includes the United States, Canada, Mexico has been introduced as the largest and most successful RTLS provider in 2015. The widespread development of the RTLS market has led to the adoption of this technology in this country. The rapid growth of this technology has led to the adoption of this system in the healthcare sector, especially in the United States (43).

Due to the awareness of the numerous advantages of real-time tracking and monitoring patients, physicians and hospital equipment, the RTLS solution market is expected to grow significantly between 2016 and 2022 (46).

The developments in the RTLS around the world represent a key trend in the field of RFID technology and innovative programs for diverse industries such as healthcare sector. The implementation of the real-time location system in the field of health is facing a lot of challenges. Some of these challenges include (a) selecting and applying appropriate systems, (b) implementation and systems integration, (c) interoperability with other systems for information management and decision-making (47). On the other hand, all real-time location systems users in healthcare facilities confirm the use of RFID in the immediate monitoring and tracking of assets & patients. Some major barriers include technological limitations, interference concerns, the high cost of implementation, lack of international standards and concern for patient privacy (48).

In addition, RFID technology requires the immediate identification and location of the entities in the healthcare centers involved in increasing the potential and reliability of the processes. Due to this, hospitals that intend to use the capabilities of RFID technologies should have a basic knowledge of the integration, compression of functions, identification of the critical requirements and common challenges of the system (49).

Some restrictions of RFID technology, the weak waves, concerns about RFID waves’ interference with other wireless waves of the equipment and the resulting negative effects on the health of the patients, medical staff, and also protecting the information security can be mentioned as some of these constraints; while the technology used in modern intelligence systems has great potential to overcome the physical constraints of the static intelligence systems, it can promote the quality of healthcare services (50).

However, RTLS is a prominent technology that improves the efficiency and effectiveness of health care systems, but real implementation should be based on site visitation, familiarity with the technology used, and precise execution schedules (5). In order to implement an applicable HRTLS, first, the main criteria of the system should be extracted in regard to the organization’s requirements and then the requirements engineering in knowledge area should be used for full coverage of the requirements (50).

HRTLS can help hospitals represent a congruent response to a disaster situation. For this reason, before implementing an HRTLS in this condition, managers must be inspected various technologies strengths & weakness points, opportunities, and threatening Challenges. According to a condensed primer: Real-time locating systems (RTLS) in healthcare, “the key to a successful HRTLS deployment lies in picking the right option(s) and solution(s) for the application(s) or problem(s)” (51).

### Limitations

One limitation of our study was that few researches about hospital real-time location systems in our country were down and we did not have enough sources for HRTLS and its requirements in Iran.

### Implications for hospital real-time location system

This paper illustrated the distinct HRTLS technological options presently available in the healthcare centers. Findings can help managers to select an HRTLS that meets their specific needs for implementation projects. The most important HRTLS policies are economic implications and patient and medical staff satisfaction. Therefore, comprehensive and applicable HRTLS based on novel technologies must be designed and implemented in Iran.

## Conclusion

The implementation of a real-time location system enables hospitals to achieve their goals such as improving efficiency, increasing patient satisfaction and reducing time and cost considerably. However, the implementation of RTLS project is facing a lot of technological challenges in Iran. Accordingly, regarding the organization’s requirements, novel technologies such as IoT and cloud computing or a combination of these two technologies can be used to design the real-time location system in order to overcome the existing constraints and shortcomings.

## Ethical Considerations

Ethical issues (including plagiarism, informed consent, misconduct, data fabrication and/or falsification, double publication, and/or submission, redundancy, etc.) have been completely observed by the authors.

## References

[1] Malik A (2009). RTLS For Dummies. Wiley Publishing, Inc, Indianapolis, Indiana.

[2] D’Souza I Ma W Notobartolo C (2011). Real-Time Location Systems for hospital emergency response. IT Professional,13(2):37–43.

[3] Costs Tags-low H, High Medium (2009). Real Time Location Systems, Clarinox Technologies Pty Ltd.

[4] Sepehri MM Vahdat D Mollabagher M (2011). Designing of business information needs system of patients with improved health care in the surgical process using RFID technology Ministry of Science, Research and Technology. Thesis: Payam Noor University of Tehran, 1. [In persian]

[5] Hong JY Suh EH Kim SJ (2009). Context-aware systems: A literature review and classification. Expert Syst Appl,36(4):8509–22.

[6] Ma XY Yang K Brayley K (2011). RTLS-based Ubiquitous Healthcare Management System Design and Implementation. White paper in RFID Journal.

[7] Carrasco VN Jackson SS (2010). Real time location systems and asset tracking: new horizons for hospitals. Biomed Instrum Technol, 44(4):318–323. 20715958 10.2345/0899-8205-44.4.318

[8] Boulos MNK Anastasiou A Bekiaris E Panou M (2011). Geo-enabled technologies for independent living: examples from four European projects. Technol Disabil, 23(1):7–17.

[9] Harrop P Holland G Das R (2017). RFID Knowledge base Case Studies. https://www.idtechex.com/research/reports/rfid_knowledgebase_case_studies_000009.asp

[10] Banchs A Serrano P Vollero L (2010). Providing service guarantees in 802.11e EDCA WLANs with legacy stations. IEEE Trans Mob Comput, 9(8):1057–1071.

[11] Tzeng SF Chen WH Pai FY (2008). Evaluating the business value of RFID: Evidence from five case studies. Int J Prod Econ, 112(2):601–613.

[12] Ilie-Zudor E Kemény Z Van Blommestein F (2011). A survey of applications and requirements of unique identification systems and RFID techniques. Comput Ind, 62(3):227–252.

[13] Hsu CC Chen JH (2011). A novel sensor-assisted RFID-based indoor tracking system for the elderly living alone. Sensors,11(11):10094–10113. 22346631 10.3390/s111110094 PMC3274273

[14] Kim SC Jeong YS Park SO (2013). RFID-based indoor location tracking to ensure the safety of the elderly in smart home environments. Pers Ubiquit Comput, 17(8):1699–1707.

[15] Pope B (2017). PresCare Deploys AeroScout Wi-Fi RFID Solution for Patient Safety and Medical Alerting. Business Wire, A Berkshire Hathaway Company. http://www.businesswire.com/news/home/20070716005284/en/PresCare-Deploys-AeroScout-Wi-Fi-RFID-Solution-Patient

[16] Bacheldor B (2007). Belgium hospital combines RFID, sensors to monitor heart patients. University Hospital of Ghent. RFID Journal. https://www.rfidjournal.com/articles/view?3120

[17] Lewis P (2007). AeroScout Wins Innovative European Healthcare Project for Patient, Staff and Asset Tracking. Business Wire (a berkshire hathaway company). https://www.eleconomista.es/empresasfinanzas/noticias/229350/06/07/AeroScout-WinsInnovative-European-Healthcare-Project-for-Patient-Staff-and-Asset-Tracking.html

[18] Bacheldor B (2008). BlueTag Patient-Tracking Comes to North America. RFID Journal. https://www.rfidjournal.com/articles/view?3906

[19] Manzoor A (2016). RFID applications in healthcare-state-of-the-art and future trends. Maximizing healthcare delivery and management through technology integration: IGI Global, 184–206. https://www.igi-global.com/chapter/RFID-applications-in-healthcare-state-of-the-art-and-future-trends/180652?camid=4v1

[20] Oztekin A Pajouh FM Delen D Swim LK (2010). An RFID network design methodology for asset tracking in healthcare. Decis Support Syst, 49(1):100–109.

[21] Alemdar H Ersoy C (2010). Wireless sensor networks for healthcare: A survey. Computer Network, 54(15):2688–2710.

[22] Vilamovska AM Hatziandreu E Schindler HR (2009). Study on the requirements and options for RFID application in healthcare. RAND technical report. https://www.rand.org/pubs/technical_reports/TR608.html

[23] Mackenzie CF Wang Y Hu PF Chen SY (2014). Automated prediction of early blood transfusion and mortality in trauma patients. J Trauma Acute Care Surg,76(6):1379–1385. 24854304 10.1097/TA.0000000000000235

[24] Delen D Erraguntla M Mayer RJ Wu CN (2011). Better management of blood supply-chain with GIS-based analytics. Ann Oper Res,185(1):181–193.

[25] Wessel R (2006). German Clinic Uses RFID to Track Blood. RFID Journal web news. https://www.rfidjournal.com/articles/view?2169/2

[26] Sini E Locatelli P Restifo N Torresani M (2009). Healthcare Professionals Identification at regional and local level: an RFID integrated scenario based on synergic experiences. Eur J Gen Pract, 1(6):39–49.

[27] Pérez MM Cabrero-Canosa M Hermida JV (2012). Application of RFID Technology in Patient Tracking and Medication Traceability in Emergency Care. J Med Syst, 36(6):3983–93. 22833319 10.1007/s10916-012-9871-x

[28] Wessel R (2006). German hospital expects RFID to eradicate drug errors. RFID Journal web news. https://www.rfidjournal.com/articles/view?2415

[29] Wessel R (2007). RFID synergy at a Netherlands hospital. RFID Journal. https://www.rfidjournal.com/purchase-access?type=Article&id=3562&r=%2Farticles%2Fview%3F3562%2F4

[30] Aziz K Tarapiah S Ismail SH Atalla S (2016). Smart real-time healthcare monitoring and tracking system using GSM/GPS technologies. https://ieeexplore.ieee.org/abstract/document/7460394

[31] Van Oranje-Nassau C Schindler HR Valeri L (2009). Study on the requierments and options for Radio Frequency Identification (RFID) application in healthcare. RAND technical report. https://www.rand.org/pubs/technical_reports/TR608z1.html?src=mobile 10.7249/rhq-01-04-05 PMC4945254 28083212

[32] Sensors Staff (2008). Hospital Uses AeroScout's Visibility Solution Questex LLC. 275 Grove Street, Suite 2-130 Newton, MA 02466. Sensors Online.

[33] Maximov V Tabarovsky O (2013). Survey of Accuracy Improvement Approaches for Tightly Coupled ToA/IMU Personal Indoor Navigation System. International Conference on Indoor Positioning and Indoor Navigation, 28th–31th October 2013.

[34] DecaWave (2017). LLC RTLS. https://www.decawave.com/llc-rtls/

[35] Yazici HJ (2014). An exploratory analysis of hospital perspectives on real time information requirements and perceived benefits of RFID technology for future adoption. Int J Inf Manage, 34(5):603–621.

[36] Park N (2011). Secure UHF/HF dual-band RFID: strategic framework approaches and application solutions. International Conference on Computational Collective Intelligence, Springer, Berlin, Heidelberg:488–496.

[37] Armstrong S Providence St (2009). Vincent Medical Center Implements Cardiovascular Unit Automatic Patient and Asset Tracking From Patient Care Technology Systems. Vice President Marketing.

[38] Fisher JA Monahan T (2012). Evaluation of Real-Time Location Systems in their hospital contexts. Int J Med Inform, 81(10):705–712. 22857790 10.1016/j.ijmedinf.2012.07.001

[39] Bourouis A Feham M Bouchachia A (2012). A new architecture of a ubiquitous health monitoring system: a prototype of cloud mobile health monitoring system. https://arxiv.org/abs/1205.6910

[40] Akiyama M (2014). Risk Management and Measuring Productivity with POAS (Point of Act System). World Congress on Medical Physics and Biomedical Engineering 2006, Springer, Berlin, Heidelberg, 1(5): 326–330.

[41] Hamitag (2014). Return on Investment on Ral-time Location System. http://www.articlebiz.com

[42] Boyd RW (2011). Location system for wireless local area network (WLAN) using RSSI and time difference of arrival (TDOA) processing. Patents Application publication. United States patent US No.7.

[43] Real-Time Locating Systems (RTLS) Market worth 8.09 Billion USD by 2022.

[44] Talebkhani R (2015). Smart Hospital, a new horizon in health care. Pars Hospital. http://www.pars-hospital.com [In persian]

[45] Real Time Location System Market-Industry Analysis, Geography, Trends, Forecast 2017-2022 (2017). Mordor Intelligence. Available at: https://www.marketsandmarkets.com/Mark et-Reports/real-time-location-systemsmarketb1322.html?gclid=EAIaIQobChMIu6rap Ous4QIVlOd3Ch3aig67EAAYASAAEgLIo _D_BwE

[46] Real-Time Locating Systems (RTLS) Market by Offering (Hardware, Software, and Service), Technology (RFID, Wi-Fi, UWB, BLE, Infrared), Application (Inventory & Asset-Tracking & Management), Vertical, and Geography-Global Forecast to 2022 (2016).

[47] Bendavid Y (2013). RFID-enabled Real-Time Location System (RTLS) to improve hospital's operations management: An up-to-date typology. Int J RF Technol, 5(3–4):137–158.

[48] Yao W Chu CH Li Z (2012). The adoption and implementation of RFID technologies in healthcare: a literature review. J Med Syst, 36(6):3507–3525. 22009254 10.1007/s10916-011-9789-8

[49] Najera P Lopez J Roman R (2011). Real-time location and inpatient care systems based on passive RFID. J NETW COMPUT Appl, 34(3):980–989. 10.1016/j.jnca.2010.04.011 PMC7148963 34170999

[50] Ajami S Sarbaz M (2014). Necessity of Usage Mobile and Wireless Communication in hospital in disasters. Health Info Manag J, 6(11):40. [In persian]

[51] Boulos MNK Berry G (2012). Real-time locating systems (RTLS) in healthcare: a condensed primer. Int J Health Geogr, 11:25. 22741760 10.1186/1476-072X-11-25 PMC3408320

